# Skin anti‐inflammatory activity of rose petal extract (*Rosa gallica*) through reduction of MAPK signaling pathway

**DOI:** 10.1002/fsn3.870

**Published:** 2018-10-25

**Authors:** Myung‐hee Lee, Tae Gyu Nam, Inil Lee, Eun Ju Shin, Ah‐ram Han, Pomjoo Lee, Sung‐Young Lee, Tae‐Gyu Lim

**Affiliations:** ^1^ Korea Food Research Institute Wanju‐gun Korea; ^2^ Department of Food Science and Biotechnology Kyung Hee University Yongin Korea; ^3^ RAFIQ Cosmetics Co., Ltd. 14 Jung‐gu Korea; ^4^ Department of Agricultural Biotechnology Seoul National University Gwanak‐gu Korea

**Keywords:** antioxidant activity, cytokine, rose petal, skin inflammation

## Abstract

The aim of this study was to investigate the skin anti‐inflammatory activity of rose petal extract (RPE) and the mechanisms underlying this phenomenon. Recently, flowers have been considered as dietary resources owing to their biological activities, such as inhibition of nephritis and hemorrhoids. The *Rosa* plant exerts various biological functions, including antioxidant and anti‐microbiological activities. Herein, we confirmed the skin anti‐inflammatory activity of RPE upon solar UV (sUV) exposure. RPE reduced sUV‐induced COX‐2 expression as well as expressions of several cytokines. Activation of MKK4‐JNK, MEK‐ERK, and MKK3‐p38 signaling pathways, which are associated with cytokine production, was also attenuated by RPE treatment. We hypothesized these RPE‐induced changes are because of its antioxidant activity, because RPE displayed drastic radical scavenging and oxygen radical absorbance capacity (ORAC). Furthermore, high anthocyanins, polyphenols, and flavonoids contents were found in RPE. Hence, these results indicated the skin anti‐inflammatory activity of RPE is because of antioxidant activity.

## 1 INTRODUCTION

Plants have been widely used for medicinal purposes since thousands of years, and there is accumulating scientific evidence regarding their biological effects (Krishnaiah, Sarbatly, & Nithyanandam, 2011; Kumar, Bhandari, Singh, & Bari, 2009; Masek, Latos, Chrzescijanska, & Zaborski, 2017; Navarro‐Gonzalez, Gonzalez‐Barrio, Garcia‐Valverde, Bautista‐Ortin, & Periago, 2015). Flowers especially have been regarded as good dietary sources owing to their various beneficial elements, such as phenolic acids, flavonols, and anthocyanins (Navarro‐Gonzalez et al., 2015); beneficial properties of flowers have been well‐defined, including antioxidant properties (Krishnaiah et al., 2011; Kumar et al., 2009; Masek et al., 2017; Mohebitabar et al., 2017). The *Rosa* (*R*.) species is widely distributed plants, and is used as a medicinal herb against nephritis and hemorrhoids (Bitis et al., 2017; Caliskan, Aka, & Oz, 2017). Because of the presence of various terpenes (glycosides), flavonoids, and anthocyanins (Knapp et al., 1998; Kumar, Bhandari, Singh, Gupta, & Kaul, 2008; Oka et al., 1998; Schieber, Mihalev, Berardini, Mollov, & Carle, 2005), roses were proposed to display diverse biological activities. Indeed, Kumar et al. (2009) reported strong antioxidant activity of methanolic extracts from *R. brunonii*,* R. bourboniana*, and *R. damascena*.

Reactive oxygen species (ROS) is produced by aberrant stimuli such as ultraviolet (UV) radiation, which can damage DNA and cellular proteins. Previous studies have demonstrated that ROS activates inflammatory signaling pathways (Chen et al., 2006; Tormos, Talens‐Visconti, Nebreda, & Sastre, 2013). We also found that *N*‐acetyl cysteine (NAC, a ROS scavenger) drastically inhibited sUV‐induced matrix metalloprotein‐1 (MMP‐1) expression (data not shown) and siRNA of p47^phox^, a subunit of NADPH oxidase (NOX); as a result, reduction of EGFR transactivation in NADPH oxidase was observed (Chen et al., 2006). ROS also mediates UV‐induced skin inflammation via activation of inflammatory signaling pathways (Choi et al., 2016; Cooper & Bowden, 2007).

Here, we demonstrated the potential of rose petal extract (RPE) on sUV‐induced skin inflammation via suppression of MAPK activation. Our results showed that RPE exerts strong antioxidant effects through cytokine suppression in epidermal cell lines exposed to sUV.

## 2 MATERIALS AND METHODS

### 2.1 Reagent

Dulbecco's modified Eagle's medium (DMEM), fetal bovine serum (FBS), penicillin–streptomycin–neomycin, and 0.5% trypsin‐EDTA were purchased from GIBCO^®^ Invitrogen (Auckland, NZ, USA). *Rosa gallica* petal was imported from Turkey through GN Bio (Gyeonggi, Korea). Specific antibodies against COX‐2, β‐actin, MKK4, JNK, MEK, ERK, MKK3, and p38 were obtained from Santa Cruz Biotech (Santa Cruz, CA, USA). Primary antibodies for p‐c‐Jun, p‐MKK4, p‐JNK, p‐MEK, p‐ERK, p‐MKK3, and p‐p38 were purchased from Cell Signaling Technology (Danvers, MA, USA). The chemiluminescence detection kit was purchased from GE Healthcare (Piscataway, NJ, USA). All other chemicals were purchased from Sigma‐Aldrich (St. Louis, MO, USA).

### 2.2 Sample preparation (rose petals extract)

Rose petals (10 g) were extracted by 70% (v/v) ethanol at 70°C for 3 hr. The extracted solution was then filtered (Whatman paper). Ethanol was subsequently evaporated, and the product was freeze‐dried.

### 2.3 Cell culture

The JB6 P+ cell line was kindly provided to us by Dr. Zigang Dong's laboratory in Hormel institute. Cells were cultured at 37°C and humidified atmosphere of 5% CO_2_ in DMEM supplemented with 10% FBS with 0.1% penicillin/streptomycin/neomycin.

### 2.4 Solar UV irradiation system

The solar UV irradiation system consists of UVA and UVB lamps. The UVA‐340 lamps were purchased from Q‐Lab Corporation (Cleveland, OH); these lamps provide optimal simulated sunlight in the critical short wavelength region from 365 to 295 nm, with a peak emission of 340 nm. The ratio of UVA to UVB was measured by a UV meter at 94.5% and 5.5%, respectively.

### 2.5 Cell viability

Cell viability was measured using the Cell Titer 96 Aqueous Solution (Promega). In brief, cells were culture in 96‐well plates and treated with RPE for 24 hr. The 100 mg/ml of RPE stock sample was treated to the cultured media as indicated concentration (50–1,000 μg/ml). Following the incubation period, 20 μl MTS solution was added, and cells were further incubated for 1 hr. Cell viability was measured with absorbance at 490 nm using a microplate reader (Infinite^®^2000 PRO, Tecan, Switzerland).

### 2.6 Total flavonoids contents

Total flavonoid content of RPE was measured using the aluminum chloride method (Jia, Tang, & Wu, 1999) with modifications, using catechin. We used RPEs with various doses of 12.5–1,000 μg/ml. Each sample of RPE (100 μl) was added to 500 μl DW, followed by the addition of 30 μl NaNO_2_. After 6 min, 60 μl AlCl_3_ was added, and the mixture was incubated for 5 min. This was followed by the addition of 200 μl of 1 M NaOH, and the final mixture was made up to 1 ml with distilled water. The solution was mixed well again and centrifuged at 1,500 *g* and 4°C for 5 min. Absorbance was measured for 200 μl supernatant at 510 nm using a multiwell plate reader (Infinite^®^2000 PRO). All experiments were performed in triplicates.

### 2.7 Total polyphenol content

Total polyphenol content of RPE was determined according to the Folin–Ciocalteu method using gallic acid. We used RPEs with various doses of 12.5–1,000 μg/ml. Each sample of RPE (10 μl) was mixed with 500 μl DW and 50 μl Folin–Ciocalteu's reagent. This was followed by the addition of 150 μl Na_2_CO_3_ (20%, W/V), and total reaction volume was made up to 1 ml with distilled water. The solution was mixed again and incubated at room temperature for 2 hr. Absorbance was measured at 765 nm using multi‐plate readers (Infinite^®^2000 PRO). All experiments were performed in triplicates.

### 2.8 Total anthocyanin content

Total anthocyanin content in RPE was measured by the pH differential method. Concentration of phenolic extracts of RPE (250, 500 μg/ml) in 0.025 M potassium chloride buffer (pH 1.0) and 0.4 M sodium acetate buffer (pH 4.5) was determined at 510 and 700 nm, respectively, following incubation at 23°C for 15 min. The anthocyanin content was expressed in mg cyanidin‐3‐glucoside (CGE)/100 L RPE. A molar absorptivity of 26,900 L/mol cm was used for cyaniding‐3‐glucoside (MW 449.2 g/mol).

### 2.9 Western blot

Following sample treatment and sUV irradiation (26 kJ/cm^2^), proteins were collected in 1× lysis buffer (Cell signaling Biotechnology, Beverly, MA). Protein concentration was determined with a dye‐binding protein assay kit (Bio‐Rad Laboratories, Hercules, CA) according to manufacturer's instructions. Equal concentrations of cellular proteins were separated on polyacrylamide gels (Bio‐Rad Laboratories), and were transferred to Immobilon P membranes (Millipore, Billerica, MA). Membranes were blocked with 5% fat‐free milk for 1 hr, and were incubated with specific primary antibodies at 4°C overnight. Proteins were hybridized with HRP‐conjugated secondary antibodies, and were visualized using the chemiluminescence detection kit (GE Healthcare, Pittsburgh, PA).

### 2.10 Cytokine array

In order to confirm changes in cytokines, we used the Mouse Cytokine Array Panel A (R&D systems™). Experiments were carried out according to the manufacturer's instruction. Briefly, cells were incubated at 37°C in humidified atmosphere of 5% CO_2_; cells were cultured in DMEM supplemented with 10% FBS and 0.1% penicillin/streptomycin/neomycin, and were treated with 400 and 800 μg/ml of RPE prior to sUV exposure. Following sUV irradiation, the media were collected, and cytokine production was determined.

### 2.11 ABTS radical scavenging assay

The ABTS radical scavenging assay was performed as previously outlined (Van Den Berg, Haenen, Van Den Berg, & Bast, 1999), with slight modification. Briefly, 0.1 M PBS (pH 7.4), 2.5 mM ABTS [2,2′‐azino‐bis (3‐ethylbenzothiazoline‐6‐sulphonic acid], and 1.0 mM AAPH [2,2′‐azobis‐2‐methyl‐propanimidamide, dihydrochloride] were mixed and incubated for 12 min in the dark at 68°C. The mixture was then quickly cooled to produce the ABTS radical solution. RPE (20 μl) and vitamin C (positive control) were added to 980 μl ABTS radical solution, and the solution was allowed to react for 10 min at 37°C. Absorbance was estimated at 734 nm using multimode microplate readers (Infinite^®^ 2000 pro).

### 2.12 DPPH radical scavenging assay

DPPH [2,2‐Diphenyl‐1‐picrylhydrazyl] radical scavenging assay was carried out as follows. Samples (0.2 ml) were added to 3 ml ethanol, to which 0.8 ml of 4 × 10^−4^ M DPPH in ethanol was added. The mixture was incubated at room temperature for 10 min, and absorbance was measured at 517 nm (Wang, Jin, & Ho, 1999). All experiments were performed in triplicates.

### 2.13 ORAC assay

The ORAC (oxygen radical absorbance capacity) assay was carried out using KOMABIOTECH (Seoul, Korea) following manufacturer's instructions. First, ORAC was estimated in fluorescence units. ORAC activity was displayed using Trolox [6‐hydroxy‐2,5,7,8‐tetramethylchromane‐2‐carboxylic acid] equivalent antioxidant capacity. Experiments were performed in triplicate.

### 2.14 Statistical analysis

All experiments were performed at least three times. Data are expressed as the mean ± *SD*. Student's *t* tests were used for single statistical comparisons. *p *<* *0.05 was used to denote statistical significance.

## 3 RESULTS

### 3.1 Rose petal extract reduces sUV‐induced cytokine production through c‐Jun inhibition

**Figure 1:**
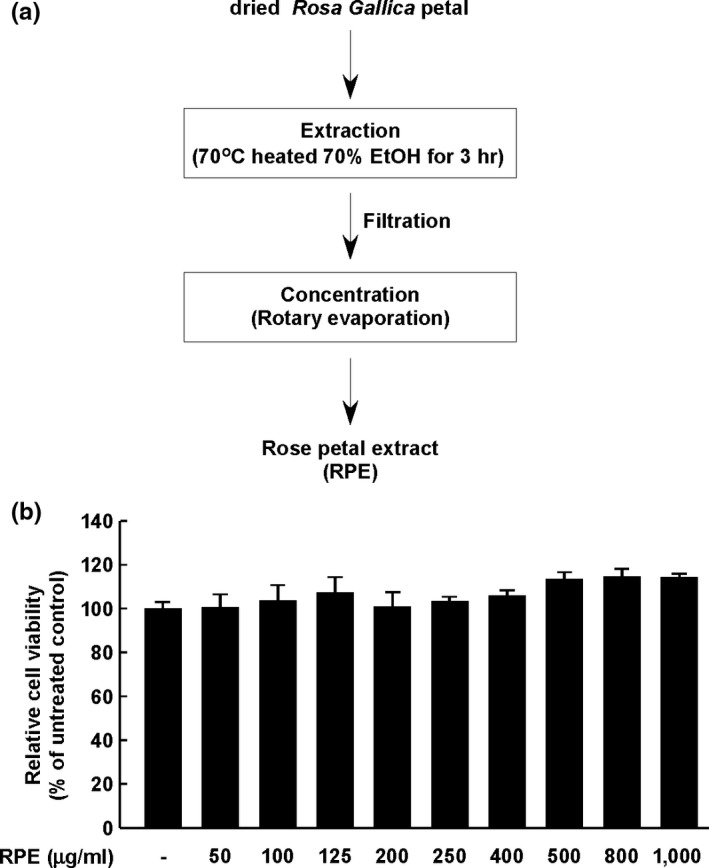
Determination of rose petal extract (RPE) cytotoxicity. Preparation of ethanol extracts from the rose petal (a). Cytotoxicity of RPE in the JB6 P+ cell line. MTS analysis was performed to determine non‐cytotoxic dose of RPE (b)

Chronic and excessive UV exposure causes skin deterioration; sUV over‐exposed skin can result in drastic clinical changes (Lopes & Mcmahon, 2016) such as skin erythema. Sunburn (erythema) is a conspicuous result of UV exposure (Matsumura & Ananthaswamy, 2004), and is accompanied by intracellular changes such as DNA damage and cytokine production (Hruza & Pentland, 1993; Lopes & Mcmahon, 2016). Pentland et al. reported that prostaglandins (PGs) are produced from arachidonic acid in UV‐irradiated human skin (Hruza & Pentland, 1993). Therefore, we investigated the effect of RPE on sUV‐induced PGs through expression of COX‐2 in the JB6 P+ cell line. RPE was obtained via the general extraction method, as shown in Figure 1a. The extract did not induce cell cytotoxicity at concentrations of ≤1,000 μg/ml in the JB6 P+ cell line (Figure 1b).

**Figure 2:**
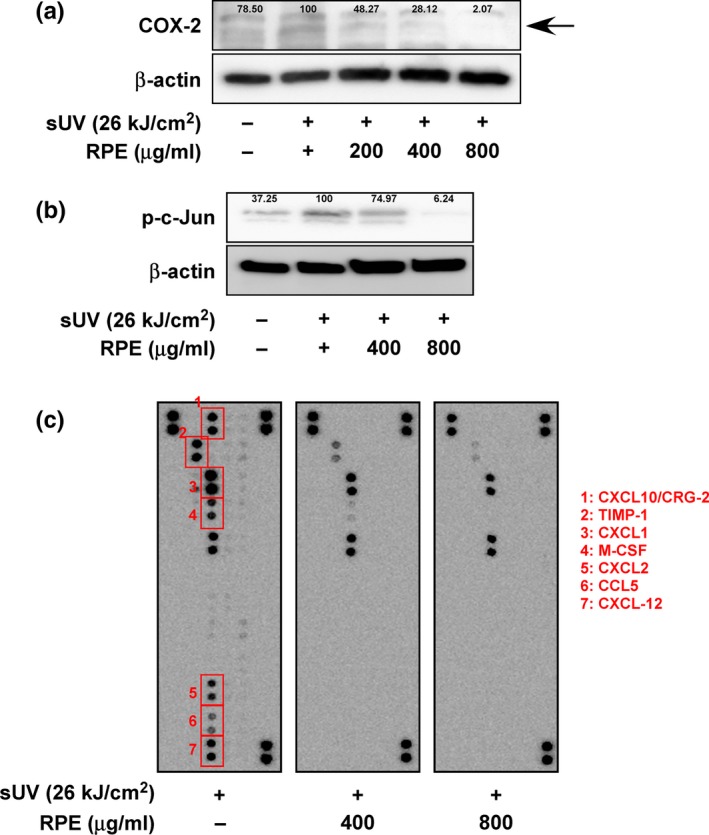
Rose petal extract (RPE) reduces sUV‐induced COX‐2 and cytokine production through c‐Jun inhibition COX‐2 expression (a) and c‐Jun phosphorylation (b) was analyzed by Western blot. The band intensity was quantified and represented as the percent of sUV‐treated control. (c) Effect of RPE on cytokine production in the JB6 P+ cell line was evaluated using Mouse Cytokine Array Panel A (*N* = 3)

As shown in Figure 2a, sUV exposure significantly elevated COX‐2 expression; 1 hr pre‐treatment of RPE resulted in dose‐dependent attenuation of sUV‐induced COX‐2 expression. We then examined phosphorylation of c‐Jun, a subunit of AP‐1. AP‐1 is a major transcription factor of the *cox‐2* gene. Similarly, phosphorylation of c‐Jun was diminished by RPE treatment (Figure 2b). Additionally, we checked the reducing activity of RPE on sUV‐enhanced cytokine production using cytokine arrays. Among the 40 different cytokines analyzed, seven cytokines showed remarkable inhibition: CXCL10/CRG‐2, TIMP‐1, CXCL1, M‐CSF, CXCL2, CCL5, and CXCL‐12 (Figure 2c).

Collectively, our results demonstrated that RPE inhibits sUV‐induced inflammatory mediator production.

### 3.2 Rose petal extract suppresses sUV‐induced MAPKK‐MAPK signaling pathway

**Figure 3:**
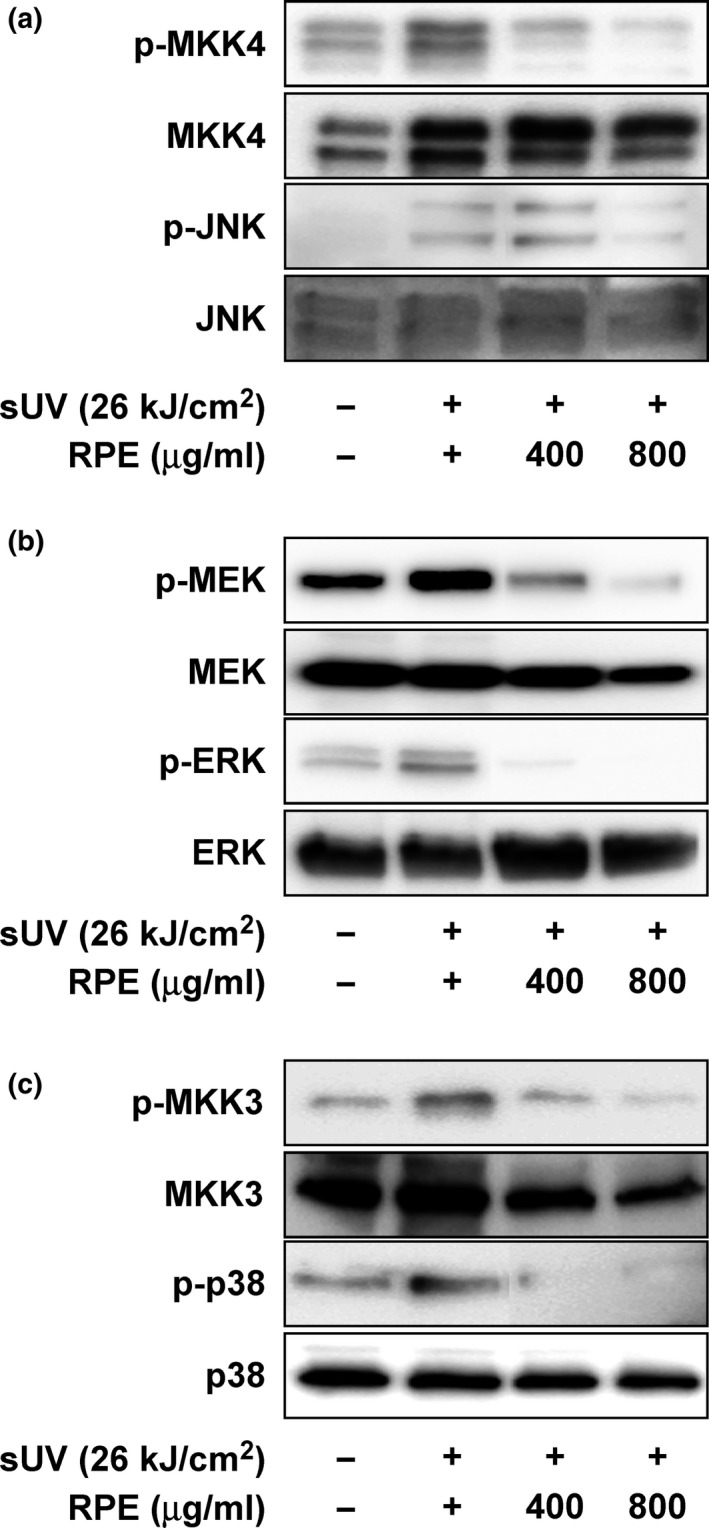
Inhibitory effect of RPE on sUV‐induced MAPKK and MAPK phosphorylation in the JB6 P+ cell line. Phosphorylation levels of MKK4‐JNK (a), MEK‐ERK (b), and MKK3‐p38 (c) were estimated by Western blot analysis using specific antibodies (*N* = 3). RPE, rose petal extract

Previous studies have associated the MAPK signaling pathway with sUV‐related skin inflammation (Hipskind & Bilbe, 1998; Reddy & Mossman, 2002). In order to confirm whether RPE affects sUV‐induced MAPK activation, we analyzed the effect of RPE on sUV‐induced MAPK signaling pathway via Western blot analysis. As shown in Figure 3, sUV irradiation significantly enhanced MKK4‐JNK, MEK‐ERK, and MKK3‐p38 signaling pathways in JB6 P+ cells. In addition, RPE pre‐treatment attenuated MAPK signaling in a dose‐dependent manner. Therefore, it was assumed that the inhibitory activity of RPE on inflammatory mediator production is mediated by suppression of the MAPK signaling pathway (Figure 2).

### 3.3 Rose petal extract display antioxidant activity

**Figure 4:**
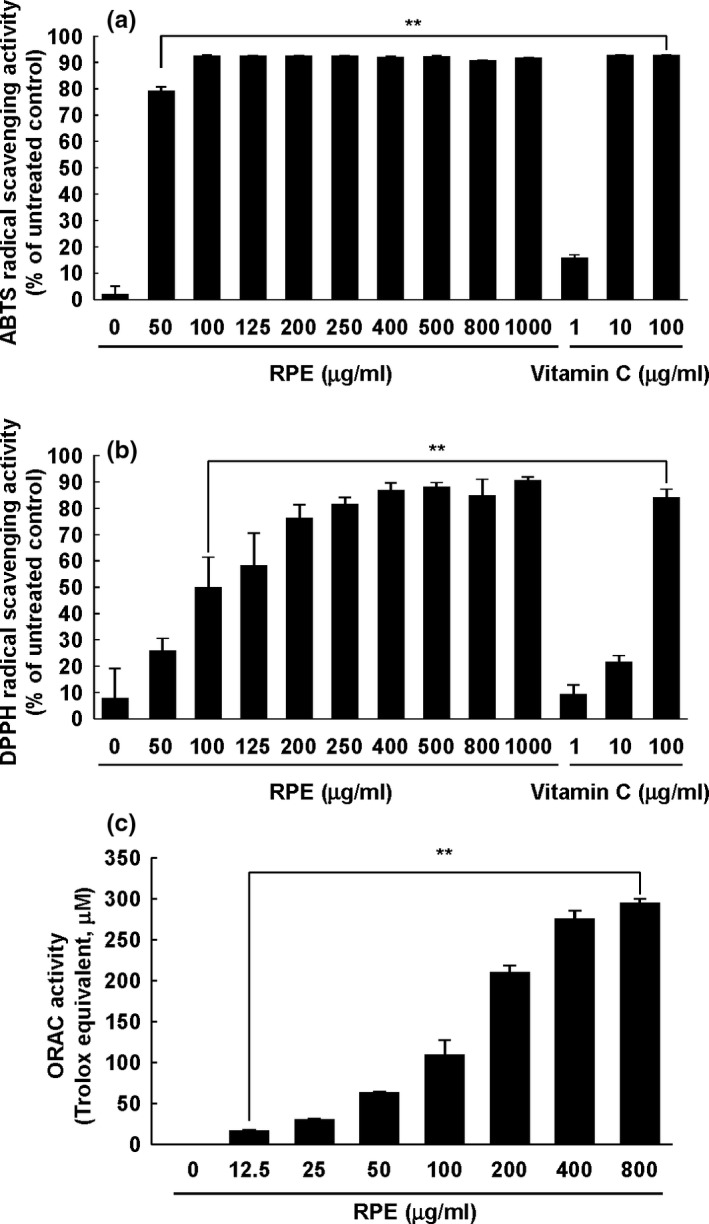
Antioxidant activity of RPE. ABTS (a) and DPPH (b) radical scavenging activity was measured. Vitamin C was used as the positive control. (c) Oxygen radical absorbance capacity (ORAC) assay was performed as described in Materials and methods. Data are representative of three independent experiments. The asterisks (**) denote statistical significance (*p *<* *0.001) as compared with controls. RPE, rose petal extract

Oxidative stress is critical for chronic human diseases such as cancer (Valko, Rhodes, Moncol, Izakovic, & Mazur, 2006). In 2006, a study reported that treatment with the ROS scavenger *N*‐acetyl cysteine (NAC) suppresses EGFR transactivation. Because EGFR is a transmembrane protein that stimulates several inflammatory signaling pathways including Ras/MAPK, we evaluated the effect of RPE on radical scavenging activity. As shown in Figures 4a and b, RPE exhibited significant ABTS and DPPH radical scavenging activity. In the case of the ABTS radical scavenging assay, nearly 80% of ABTS radicals were inhibited by 50 μg/ml RPE, which is a relative low dose (Figure 4a). To obtain more reliable antioxidant activity measurements of RPE, we tested the ORAC of RPE. As seen in Figure 4c, RPE presented dose‐dependent oxygen radical capacity.

### 3.4 Rose petal extract contains high amount of anthocyanins/flavonoid/polyphenols

**Figure 5:**
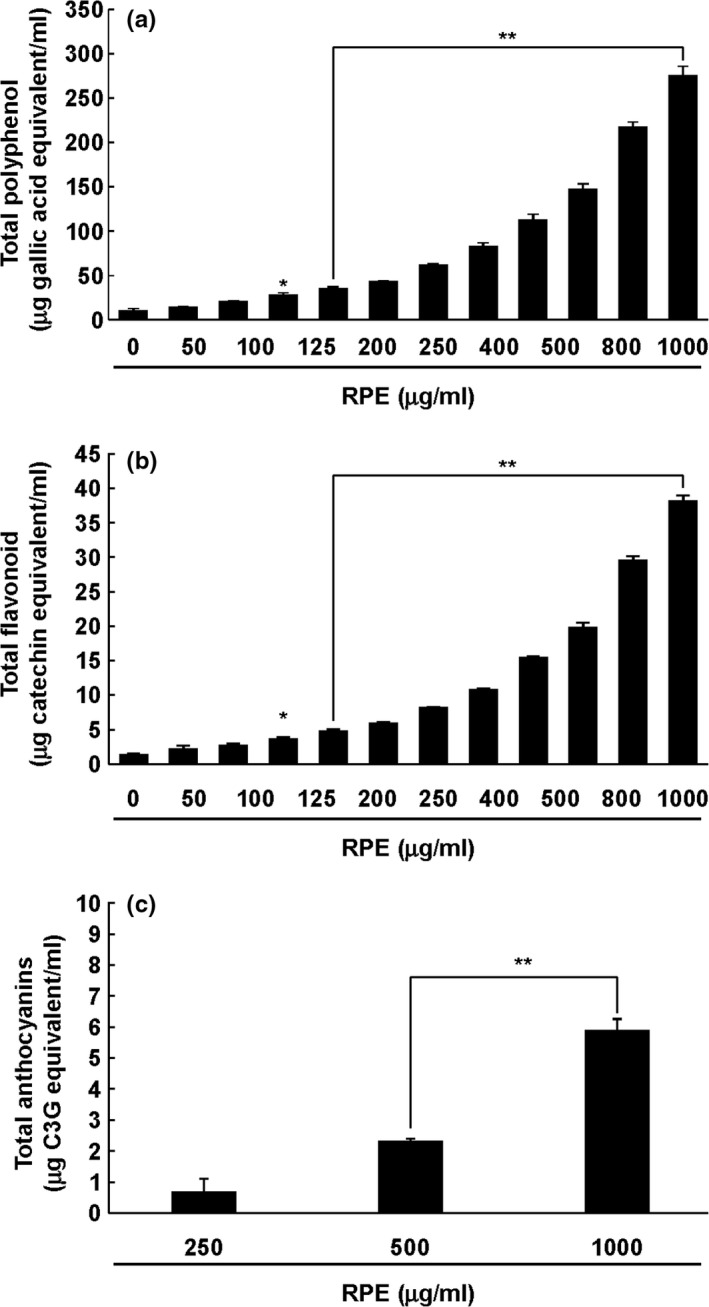
Anthocyanins/flavonoid/polyphenol content in RPE. Polyphenol (a), flavonoid (b), and anthocyanins (c) content in RPE. Gallic acid (a), catechin (b), and C3G (c) were used as standards in the assay (*N* = 3). The asterisks (* and **) indicate statistical significance (*p *<* *0.01 and 0.001) as compared with controls. RPE, rose petal extract

As it is well‐known that rose petals possess various polyphenols (Knapp et al., 1998; Kumar et al., 2008; Oka et al., 1998; Schieber et al., 2005), we determined the content of polyphenol, flavonoids, and anthocyanins in RPE. Polyphenol content was assayed using Folin–Ciocalteu's reagent. As shown in Figure 5a, dose‐dependent increase in polyphenols was observed with increased concentration of RPE. In particular, concentration of flavonoid was high in RPE (Figure 5b). Anthocyanins are known as natural oxidation suppressants associated with inflammation (Amini, Muzs, Spencer, & Yaqoob, 2017; Kim, Han, Ha, & Kim, 2017). Therefore, we verified the amount of anthocyanins in RPE. As shown in Figure 5c, nearly 6 μg C3G equivalent/ml of anthocyanins was found in 1,000 μg/ml RPE.

## 4 DISCUSSION

Flowers have been regarded not only as ornaments, but also edible plants owing to their nutritional and functional importance. Many studies have attempted to unveil the molecular actions and mechanisms of edible flowers (Caliskan et al., 2017; Knapp et al., 1998; Kumar et al., 2008; Oka et al., 1998; Schieber et al., 2005). Flowers are good sources of beneficial phenolic compounds including anthocyanins (Navarro‐Gonzalez et al., 2015). Anthocyanin is a naturally occurring pigment in fruits and flowers. Several previous reports have illustrated the beneficial activities of anthocyanin (Kwon et al., 2009; Matsumoto et al., 2001; Meiers et al., 2001).

We investigated the skin anti‐inflammatory activity of RPE in the current study. We adopted the excessive sUV exposure‐related skin inflammation model, as over‐exposure of skin to UV is a physiological stimulator of skin inflammation (Lopes & Mcmahon, 2016). The JB6 P+ cell line is an optimized model for studying skin inflammation owing to its sensitivity to inflammatory enhancers such as UV, EGFR, and 12‐O‐tetradecanoylphorbol‐13‐acetate (TPA) (Byum et al., 2013; Chen et al., 2001). We found that COX‐2 expression induced by sUV irradiation is dose‐dependently attenuated by RPE treatment. In addition, as per cytokine array results, production of several cytokines was reduced by RPE pre‐treatment (Figure 2). Furthermore, cellular signaling pathways associated with production of COX‐2 and other cytokines were down‐regulated by RPE pre‐treatment. Our results found that inhibition of MKK4‐JNK, MEK‐ERK, and MKK3‐p38 is an underlying mechanism of RPE‐mediated suppression of sUV‐induced cytokine production.

The antioxidant activity of the *Rosa* species is well‐reported in previous literatures (Kumar et al., 2009; Masek et al., 2017; Navarro‐Gonzalez et al., 2015). Similar to previous studies, we found that RPE possesses strong antioxidant effects (Figure 4). As demonstrated by the ABTS assay (Figure 4b), 100 μg/ml RPE exhibited over 90% ABTS radical scavenging activity; this was comparable to that of 10 μg/ml vitamin C (Figure 4a). As we described in the introduction section, aberrant ROS production can accelerate DNA damage and activate inflammatory signaling pathways (Chen et al., 2006; Tormos et al., 2013). Therefore, we hypothesized that suppression of MAPK signaling pathway is mediated by antioxidant activities of RPE.

**Figure 6:**
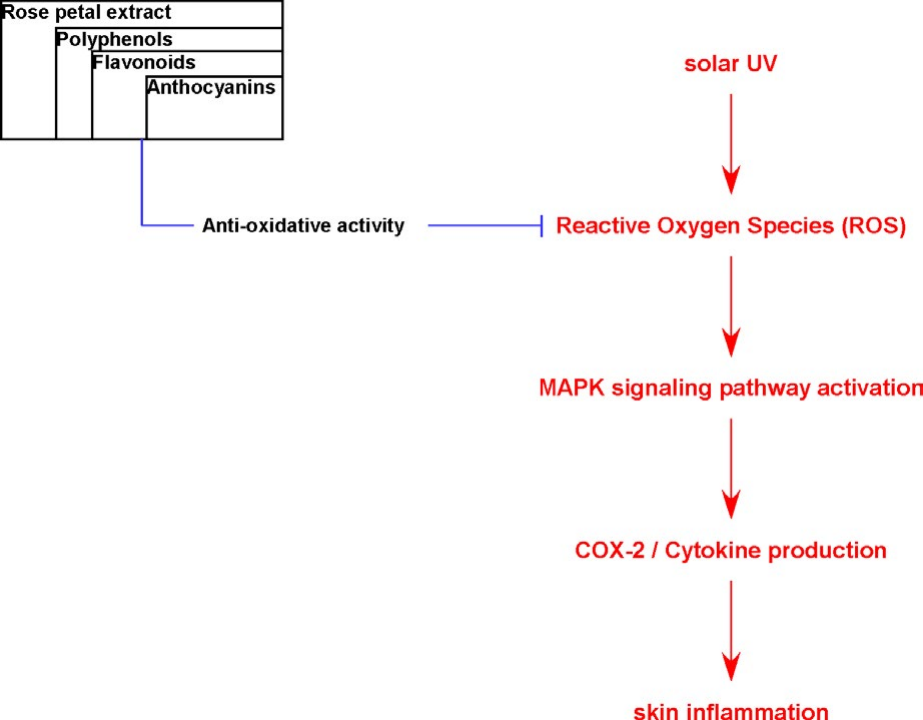
Proposed mechanism of action of RPE on sUV‐induced COX‐2 expression. RPE, rose petal extract

While the bioactive chemical in RPE is yet to be identified, the present study suggests that 70% ethanol extract from rose petals exhibit skin anti‐inflammatory and antioxidant activities via MAPK inactivation This is the first study that confirmed anti‐skin inflammatory effects of RPE. Especially, we found that RPE possess anthocyanins which are well‐known anti‐oxidative chemicals among of polyphenols. Hence, we assumed that anthocyanins in RPE showed skin anti‐inflammatory activity by their anti‐oxidative effect (Figure 6).

## 5 CONCLUSION

We acquired ethanol extracts from the petals of *R. gallica*. RPE showed anti‐skin inflammatory effects through suppression sUV‐induced MAPK activation. Based on these findings, we have showcased the potential of RPE in novel applications.

## ACKNOWLEDGMENTS

This research was supported by Main Research Program (E0183112‐01) of the Korea Food Research Institute (KFRI) funded by the Ministry of Science and ICT and supported by the Korea Institute of Planning and Evaluation for Technology in Food, Agriculture, Forestry and Fisheries (IPET) through High Value‐added Food Technology Development Program, funded by Ministry of Agriculture, Food and Rural Affairs (MAFRA) (116030‐3).

## CONFLICT OF INTEREST

The authors declared that they have no conflict of interest.
